# Ten Years of Laparoscopic Pectopexy: A Case Series Analysis

**DOI:** 10.1007/s00192-025-06377-7

**Published:** 2025-11-03

**Authors:** Guenter Noé, Elisavet Farsaki, Michael Anapolski, Anna Pitsillidi

**Affiliations:** 1 Department of OB/GYN, University of Witten Herdecke, 58448 Witten, Germany; 2 Department of OB/GYN, Rheinland Klinikum Dormagen, Dr. Geldmacher-Strasse 20, 41540 Dormagen, Germany; 3 Department of OB/GYN Agaplesion, Bethesda Krankenhaus Wuppertal, Hainstrasse 35, 42109 Wuppertal, Germany

**Keywords:** Pelvic organ prolapse, Pelvic floor disorders, Recurrence, Reconstructive surgical procedures, Laparoscopy

## Abstract

**Introduction and Hypothesis:**

Pelvic organ prolapse (POP) often causes significant urinary, bowel, pain, and sexual symptoms, and treatment success is increasingly defined by symptom relief rather than anatomical correction alone. Long-term outcome data for laparoscopic pectopexy (LP) remain limited.

**Methods:**

We conducted a retrospective cohort study of all women undergoing multicompartment POP repair with LP and native tissue repair of level 2 and 3 defects at a tertiary referral center between 2010 and 2019. The primary outcome was prolapse recurrence, defined as POP-Q stage II or higher. Secondary outcomes included de novo POP, surgical reoperation, and complications. Time-to-event outcomes were analyzed with Kaplan–Meier survival curves to estimate freedom from recurrence and freedom from reoperation.

**Results:**

A total of 832 patients were included (median age of 63 years). Median recurrence-free survival was 9.34 years (IQR 7.36–10.60), and median reoperation-free survival was 9.56 years (IQR 7.68–10.71). Most events (87.9%) occurred within 4 years. Surgical reoperation was required in 81 patients (9.7%), also clustering in the first 4 years. Apical re-fixation achieved a 94.9% long-term success rate over 10 years. Logistic regression identified lateral defect severity as the strongest predictor of failure (*p* < 0.001), whereas demographic factors and prior surgery had minimal impact.

**Conclusions:**

LP with defect-oriented native tissue repair provides durable apical support with a low 10-year reoperation rate. Most clinically relevant failures occur within 4 years, supporting follow-up of this duration. Addressing lateral defects during initial surgery is critical for optimizing long-term outcomes.

**Supplementary Information:**

The online version contains supplementary material available at 10.1007/s00192-025-06377-7.

## Introduction

Pelvic floor defects (PFD) show a wide range of symptoms. These vary from pure pressure complaints to bladder emptying disorders, defecation problems, pain and sexual disorders. The restrictions on everyday life can be massive. For classification, a consensus of IUGA and ICS provides a tool for daily practice [1, 2]. There is now a widespread consensus that the assessment of treatment success focuses more on the symptoms rather than exclusively on the anatomical results, especially to prevent unnecessary second interventions [3–5].

The development of surgical pelvic floor treatment is subject to continuous development, but there is still a lack of long-term data to guide planning a surgical strategy. Although long-term follow-up can provide valuable insights, previous studies have shown that attrition rates increase substantially after 3–5 years in older populations due to comorbidities, reduced mobility, or lack of symptoms [6]. Moreover, large registry-based analyses indicate that the majority of clinically significant pelvic organ prolapse recurrences—and reoperations—occur within the first 3–5 years after surgery, with late failures being relatively uncommon [7]. As a result, 10-year follow-up in elderly surgical cohorts often relies on registry data or retrospective chart reviews rather than consistent physical examinations [8, 9]. In our own cohort, many patients were already of advanced age at the time of their initial surgery, and asymptomatic individuals were less likely to attend late follow-up visits, even if enrolled in studies.

The discussion about the application of mesh materials is very active globally and the search for the best treatment continues. For 20 years, we have been using the combination of the treatment of levels 2 and 3 in native tissue repair in combination with apical fixation by mesh material (sacropexy/pectopexy). This allows for less mesh use [10, 11]. Since the intermediate results are convincing, we followed up a cohort that underwent surgery in 9 years over a period of 4–10 years after initial surgery.

## Materials and Methods

We performed a retrospective cohort study (approval number 173/19) including all women who underwent multicompartment pelvic organ prolapse (POP) surgery at our tertiary referral center between January 2010 and December 2019. All procedures incorporated apical fixation using laparoscopic pectopexy (LP) with concomitant native tissue repair of level 2 and 3 defects.

The primary outcome was prolapse recurrence, defined as POP-Q stage II or higher in any compartment previously treated. De novo POP was defined as new prolapse (POP-Q stage II or higher) in a compartment not involved or treated during the primary surgery. Secondary outcomes were de novo prolapse, surgical reoperation for POP (recurrence or de novo), and perioperative complications.

All relevant data, including demographic characteristics, obstetric history, surgical details, POP-Q staging, postoperative symptoms, and subsequent interventions, were retrieved from electronic medical records.

### Patient Population

A total of 832 patients who underwent POP surgery at our clinic between 2010 and 2019 were registered in the clinic database. Inclusion criteria encompassed patients who underwent LP using a mesh (PRP 3 × 15 Dynamesh) either as a stand-alone procedure or in combination with level 2 and 3 native tissue corrections. LP was performed either with concomitant supracervical hysterectomy or on the vaginal vault in patients who had already undergone a total hysterectomy. In all cases, native tissue repairs for level 2 and 3 defects were performed vaginally at the beginning of the procedure, prior to the laparoscopic part. In our unit, cystoceles are routinely classified as midline or lateral defects. When a lateral defect was clinically dominant—most often in younger patients—we performed a laparoscopic lateral defect repair using a suture technique. A combined vaginal colporrhaphy with lateral repair was rarely required and was not included in the statistical analysis. The presence and severity of a lateral defect were documented for all patients and considered in the risk-factor evaluation.

Demographic data collected included patient age, BMI, parity (number of vaginal deliveries and cesarean sections), and relevant medical history. Prior surgeries were defined as any previous pelvic or gynecological procedures, such as hysterectomy, prolapse repair, or incontinence surgeries.

The Pelvic Organ Prolapse Quantification (POP-Q) system following IUGA/ICS recommendations [12]. While POP-Q measurements were used for staging, lateral defects were identified clinically through site-specific assessment during POP-Q examination. Key indicators of lateral (paravaginal) defects included asymmetrical anterior wall descent, widened vaginal sulci, and palpable loss of lateral support, without central bulging [13]. Lateral defect severity was classified on the basis of intraoperative and POP-Q examination findings as mild (paravaginal descent < 1 cm above the ischial spine), moderate (at ischial spine level), or severe (> 1 cm below ischial spine level). A detailed review of secondary or tertiary surgical procedures was performed, and both complications and general clinical symptoms were documented. A distinction was made between de novo defects and recurrent prolapse. For the purpose of this study, prolapse recurrence was defined as the reappearance of POP in a previously corrected compartment reaching POP-Q stage II or higher, whereas a de novo defect referred to the development of new prolapse (POP-Q stage II or higher) in a compartment not involved or treated during the primary surgery. Given the strong relationship with referring physicians and the high connection to our patient population, follow-up was facilitated with a low rate of patient loss to follow-up.

### Data Collection

All relevant data, including demographic characteristics, surgical details, preoperative and postoperative symptoms, POP-Q stage of prolapse, and the occurrence of recurrent or de novo defects, were extracted from the clinic’s electronic records. Data on secondary or tertiary surgical procedures, complications, and other clinical outcomes were also recorded.

### Statistical Analysis

Descriptive statistics were used to summarize patient characteristics. Means and standard deviations (SD) were calculated for normally distributed continuous variables, while medians and interquartile ranges (IQR) were used for non-normally distributed variables. Frequencies and percentages were reported for categorical variables. The Mann–Whitney U test was used for comparisons of non-normally distributed continuous variables, and Pearson’s chi-square or Fisher’s exact test for categorical variables. Multivariate logistic regression identified predictors of prolapse recurrence and surgical reintervention, and results are presented as odds ratios (OR) with 95% confidence intervals (CI).

For patients without an event, the last documented clinic or electronic record contact was used as the censoring date. Time zero was the date of primary surgery. Kaplan–Meier survival analysis estimated (1) freedom from prolapse recurrence (including de novo prolapse, POP-Q ≥ II) and (2) freedom from surgical reoperation for prolapse. Separate survival curves were plotted on a single figure with annual numbers at risk shown below the x-axis. Survival distributions were compared descriptively; no formal log-rank comparison was required. All analyses were conducted using IBM SPSS Statistics for Windows, version 29.0 (IBM Corp., Armonk, NY, USA), with statistical significance set at *p* < 0.05.

## Results

### Descriptives of the Total Population

This study included 832 participants with a median age of 63 years (IQR 52–72) and a median BMI of 25.7 kg/m^2^ (IQR 23.2–29.0). Participants had a median of 2 vaginal deliveries (IQR 1–2) and 0 caesarean sections (IQR 0–0) (Table 1). Most patients (510) had no prior gynecological surgery; among those who did, the most common were vaginal hysterectomy (121), anterior colporrhaphy (72), and laparoscopic supracervical hysterectomy (LSH) (70) (Supplementary Table 1). Preoperative staging according to POP-Q showed that most patients had stage II central (apical) prolapse (60.6%), followed by stage III (24.8%) and a few stage IV. Anterior compartment prolapse was stage II in 22.0% and stage III in 18.6% of patients. Posterior compartment prolapse was stage II in 35.8% and stage III in 6.5%. Lateral defects, evaluated clinically using the same stage definitions as POP-Q but not part of the formal system, were present as stage II in 34.0% and stage III in 10.7% of patients (Supplementary Table 2). All patients underwent LP alone or with level 2/3 tissue correction. Common concomitant surgeries included salpingectomy, posterior colporrhaphy, and anterior colporrhaphy (Supplementary Table 3).

**Table 1:** Descriptive statistics of participant characteristics

	Median (IQR)
Age (years)	63 (52–72)
Height (cm)	165 (160–170)
Weight (kg)	70 (63–80)
BMI (kg/m^2^)	25.7 (23.2–29.0)
Vaginal deliveries	2 (1–2)
Caesarean sections	0 (0–0)

### Failure Events: Recurrence and Reoperation

The primary outcome was the incidence of prolapse recurrence or de novo prolapse after LP, performed alone or in combination with level 2 and 3 native tissue repairs. Among 832 patients, 83.2% (*n* = 692) had no recurrence or new prolapse, while 16.8% (*n* = 140) experienced recurrence or de novo POP. This subgroup had a median age of 63 years (IQR 52–72) and a median BMI of 25.4 kg/m^2^ (IQR 23.1–29.3), with a median of 2 vaginal deliveries (IQR 1–2) and 0 caesarean sections (IQR 0–0) (Supplementary Table 4). Most patients with recurrence/de novo POP (95/140) had no prior gynecological surgery; the remaining 45 had undergone procedures, including laparoscopic supracervical hysterectomy (*n* = 15), abdominal hysterectomy (*n* = 10), vaginal hysterectomy (*n* = 10), anterior colporrhaphy (*n* = 11), posterior colporrhaphy (*n* = 7), midurethral sling (TVT/TOT; *n* = 4), laparoscopic colposuspension (*n* = 2), or other prolapse surgeries (*n* = 10) (Supplementary Table 5).

Preoperative POP-Q staging showed apical prolapse was moderate in 62.9%, severe in 29.3%, and maximal in 4.3%. Posterior prolapse was mild to moderate in 57.8% and severe in 8.6%. Anterior prolapse was absent in 47.1%, mild to moderate in 30%, and severe in 22.9%. Lateral defects were moderate in 38.6% and severe in 22.1% (Supplementary Table 6).

**Fig. 1:**
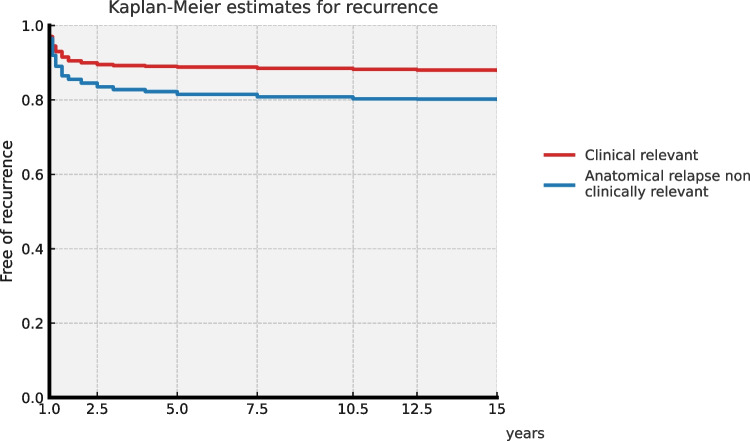
Kaplan–Meier curves showing freedom from prolapse recurrence (blue) and freedom from surgical reoperation-clinical relevant (orange) during 10 years of follow-up after LP

Kaplan–Meier survival analysis was used to estimate the probabilities of remaining free from prolapse recurrence and from surgical reoperation, with the date of surgery as time zero and censoring at the last documented clinical contact. The median recurrence-free interval was 9.34 years (IQR 7.36–10.60), and the median reoperation-free interval was 9.56 years (IQR 7.68–10.71). Both curves showed a steep early decline, indicating that most recurrences and reoperations occurred within the first 4 years after surgery, followed by a prolonged plateau reflecting a very low risk of late events (Fig. 1).

Compartment-specific recurrence and de novo prolapse rates were low when expressed relative to the total cohort (*n* = 832). Apical recurrence occurred in 8.0% of patients, while anterior and posterior compartment recurrences were observed in 2.0% and 2.2%, respectively. Lateral defect recurrence was uncommon (1.3%). De novo prolapse affected 6.0% anteriorly, 4.3% posteriorly, and 3.0% laterally. Detailed compartment-specific distributions are provided in Supplementary Table 7.

Over the 10-year follow-up, 81 patients (9.7%) required surgical reoperation, most occurring within the first 4 years. Apical re-fixation was performed in 5.05% (*n* = 42), yielding a long-term apical cure rate of 94.9%. An additional 5.0% (*n* = 41) had apical relapse and 0.6% (*n* = 5) had a second apical recurrence. Posterior colporrhaphy was performed for recurrent rectocele in 2.4% (*n* = 20) and for de novo rectocele in 4.4% (*n* = 37). Midline cystocele recurrence occurred in 2.2% (*n* = 18) and de novo cystocele in 6.9% (*n* = 57). Lateral defect recurrence was rare (1.3%, *n* = 11), and de novo lateral defects or stress urinary incontinence (SUI) occurred in 3.0% (*n* = 25).

### Association Between Patient Characteristics and Recurrence or De Novo Prolapse

We analyzed demographic, clinical, and prolapse-related factors for associations with recurrence and the need for reoperation or treatment of de novo prolapse. Most variables—including age, BMI, obstetric history, and compartment-specific prolapse severity at uterine, cervical, vaginal vault, anterior, and posterior sites—showed no significant differences between patients with and without recurrence. However, lateral defect severity was significantly higher in those with recurrence or new prolapse (median 2 vs. 1; *p* < 0.001) (Table 2).

**Table 2:** Comparison of characteristics between recurrence/de novo defects (*n* = 140) and no recurrence/de novo defects (*n* = 692) groups

Variable	Median (no recurrence)	IQR (no recurrence)	Median (recurrence)	IQR (recurrence)	Mann–Whitney U	*p* value
Age (years)	62.5	19.75	63.0	19.25	46,891	0.586
BMI (kg/m^2^)	25.79	5.75	25.43	5.96	43,506	0.611
Number of vaginal deliveries	2	1.00	2	1.00	43,761.5	0.620
Number of caesarean sections	0	0.00	0	0.00	42,167	0.182
Uterine, cervical or vaginal vault prolapse	2	1.00	2	1.00	52,720	0.059
Posterior vaginal wall prolapse	1	2.00	1	2.00	48,411.5	0.991
Anterior vaginal wall prolapse	1	2.00	1	2.00	48,604.5	0.946
Lateral defect	1	2.00	2	2.00	60,125.5	< 0.001
Overall POPQ stage (n, %)						0.135^1^
Stage II	339 (49.0%)	–	60 (42.9%)	–		
Stage III	290 (41.9%)	–	69 (49.3%)	–		
Stage IV	45 (6.5%)	–	11 (7.9%)	–		
Stage I	17 (2.5%)	–	0 (0%)	–		

^1^ Fisher’s exact test for grouped comparison (stage II vs III–IV), *IQR* Interquartile range; *p*: *p* value

When assessing overall preoperative POP-Q stage, recurrence was more frequent among patients with advanced prolapse (stage III–IV) compared to those with stage II (57.1% vs. 48.4%). However, this association was not statistically significant (Fisher’s exact test, *p* = 0.135). Logistic regression analysis confirmed a similar trend, with advanced stage showing a higher, but non-significant, risk of recurrence (OR 1.41, 95% CI 0.98–2.06, *p* = 0.065). Although there was a tendency toward higher recurrence with advanced stage, the predictive value of overall stage was limited, and stage alone did not emerge as an independent risk factor. Prior gynecological surgery was also not significantly linked to recurrence or de novo prolapse (*p* = 0.120).

Additionally, we specifically investigated the association between apical prolapse recurrence and patient characteristics. Apical recurrence was also strongly linked to higher lateral defect severity (*p* < 0.001), but not to age, BMI, obstetric history, or other prolapse compartments (Supplementary Table 8). Prior surgeries showed no significant correlation with apical recurrence (*p* = 0.070).

Reoperation at first follow-up was significantly associated with more severe lateral defects (median 2 vs. 1; *p* < 0.001) but not with other variables (Supplementary Table 9). Gynecological surgery history was not significantly linked (*p* = 0.188). For de novo defect surgery, only lateral defect severity was a significant predictor (*p* < 0.001) (Supplementary Table 10). Interestingly, patients without prior surgeries were more likely to require surgery (*p* = 0.024).

Regarding apical prolapse, different types of hysterectomy were analyzed for their association with apical recurrence at the first follow-up. No significant links were found between apical recurrence and laparoscopic supracervical, abdominal, total laparoscopic, or abdominal supracervical hysterectomies. However, vaginal hysterectomy was significantly associated with a lower risk of apical recurrence (*p* = 0.003) (Supplementary Table 11).

Across multiple logistic regression models, lateral defect severity was the strongest predictor of prolapse recurrence (OR 1.55), reoperation (OR 1.79), and overall surgical intervention (OR 1.94). Traditional factors such as age, BMI, and delivery history showed no significant associations, except BMI had a marginal link to surgery need (OR 1.07). Vaginal hysterectomy was linked to a significantly lower risk of apical recurrence (OR 0.09), and prior gynecological surgeries were associated with reduced odds of surgery at follow-up (OR 0.49) (Supplementary Tables 12–15).

## Discussion

### Recurrence and De Novo Defects

This 10-year retrospective study confirms the long-term durability of LP with combined defect-oriented native tissue repair at levels 2 and 3. Only 9.7% of patients (81) required additional surgical treatment over the full follow-up, and 85% of recurrences occurred within the first 4 years. The low long-term reoperation rate and sustained apical cure rate (94.95%) demonstrate that LP provides stable anatomical support. Kaplan–Meier survival curves reinforce this pattern, showing that the risk of both recurrence and reoperation is concentrated in the early postoperative years and remains very low thereafter, underscoring the long-term durability of the repair.

Although our study did not include a sacrocolpopexy (SCP) comparator, published long-term SCP series help contextualize these results. The PFDN/CARE extension of open abdominal SCP (~7 years) reported estimated anatomic failure rates of 22–27%, symptomatic failure of 24–29%, and mesh erosion of ~10.5% [14]. Similarly, a prospective laparoscopic SCP cohort with a median follow-up of 85.5 months reported 8.6% apical recurrence and 17.8% overall reoperation (including 7.0% graft-related procedures) [15]. By contrast, LP in our series used mesh only for apical fixation, avoiding level-2 mesh placement and the associated erosion risk at that site. While not a direct comparison, these findings suggest that limiting mesh to apical support may offer a favorable long-term risk–benefit profile.

Our findings align with Noé et al., who reported lower apical relapse rates with LP compared to laparoscopic sacropexy (LS) (2.3% vs. 9.8%) [10], and with an international multicenter trial reporting a comparable apical cure rate of 94.3% [16]. However, the present study provides substantially longer follow-up and a much larger patient cohort. Differences in reported recurrence rates across studies may reflect variations in surgical approach, patient selection, and follow-up duration. In particular, our consistent distinction between lateral and central cystoceles—an aspect rarely addressed in other research—may partly explain our lower observed recurrence rates for certain compartments.

There are few studies examining large patient cohorts over extended periods [6, 7], making our data particularly relevant. In our series, patients in levels 2 and 3 were treated exclusively with native tissue repair, while synthetic mesh was used only for level 1 procedures. The fact that these mesh-sparing repairs achieved outcomes comparable to mesh-augmented alternatives, such as Y-mesh sacropexy [9], is noteworthy and supports the effectiveness of our approach.

Khoiwal et al. reported an apical recurrence rate of 6.7% in both the LP and LS groups, without observing de novo prolapse in other compartments. However, this randomized control trial (RCT) was limited by its short follow-up period (6 months) and small sample size (15 patients per group) [17]. Obut et al. similarly reported a 3.2% apical relapse rate in the LP group after 12 months, with no recurrences in the LS group [18].

The anterior vaginal wall was the most frequent site of non-apical recurrence, consistent with prior literature [19]. However, most of these cases did not require surgical intervention, indicating that anatomical changes in non-apical compartments may not always translate into clinical burden.

### Associated Factors for Recurrence or De Novo Defects

Despite significant progress in reconstructive surgical techniques, POP remains prone to recurrence. The limited understanding of associated factors complicates the identification of patients who might benefit from adjunctive measures such as mesh-augmented repairs.

Our study revealed no statistically significant differences in baseline characteristics—including age, BMI, parity, and obstetric history—between patients with and without prolapse recurrence or de novo prolapse. Similarly, Boder-Adler et al. found no overall correlation with baseline characteristics; however, they did observe that patients with recurrence were significantly older [20]. On the other hand, Manodoro et al. identified obesity, severe macrosomia and premenopausal status as potential associated factors for POP recurrence [21]. In the present study, only a marginal association could be detected between BMI and the need for surgical intervention.

Preoperative overall POP-Q stage, often cited as a robust predictor of recurrence—particularly stage III or IV disease [20–24]—did not show a statistically significant relationship with long-term outcomes in our analysis (Fisher’s exact test *p* = 0.135; logistic regression OR 1.41, 95% CI 0.98–2.06, *p* = 0.065). In contrast, prior laparoscopic series have demonstrated a clear association between advanced preoperative POP-Q stage and an increased risk of recurrence. For example, Aslam et al. reported 3.8-fold increased odds of recurrence with higher preoperative stage in patients undergoing sacrocolpopexy [25] and Sato et al. found a 3.5-fold increased risk specifically associated with stage IV disease [26]. The difference in our study may reflect the advantages of combining laparoscopic pectopexy with native tissue repair to address site-specific defects, potentially mitigating the impact of stage alone. Clinically, this suggests that while higher stage should raise awareness of recurrence risk, surgical decision-making should also consider the underlying defect pattern and repair strategy rather than stage alone.

Furthermore, contrary to findings by Shi et al., we did not observe an association between prior gynecologic surgery and increased risk of recurrence or de novo defects [23]. Intriguingly, previous surgeries appeared to reduce the likelihood of requiring reoperation for de novo prolapse, potentially reflecting altered tissue characteristics or surgical access. Additionally, a reduced apical recurrence rate was noted when mesh fixation was performed at the vaginal cuff rather than the cervix, where loosening and necrosis were implicated in failure. However, further studies are needed to explore these associations and their underlying mechanisms in greater detail.

A particularly robust and clinically relevant finding was the strong association between lateral defect severity and unfavorable postoperative outcomes. Patients with higher stage lateral defects consistently exhibited increased overall and apical recurrence rates and a greater need for reoperation (*p* < 0.001). These findings may suggest that simultaneous correction of lateral defects—even in asymptomatic patients—could be beneficial in reducing the risk of recurrence and the need for additional surgery. This is supported by Oversand et al., who demonstrated that addressing all compartments lowered long-term reoperation rates [8]. Moreover, Manodoro et al. found that omission of posterior repair increased recurrence risk across compartments, while anterior repair showed a trend toward higher recurrence rates [21].

Collectively, this evidence supports the notion that incomplete repair—particularly of lateral and posterior compartments—may predispose patients to prolapse recurrence. A more comprehensive surgical strategy addressing all anatomical defects may thus offer improved long-term outcomes.

An additional point of consideration is the choice of apical support strategy. While sacrospinous ligament fixation (SSLF) and uterosacral ligament suspension (USLS) are widely used alternatives, long-term outcome data remain limited and heterogeneous. The OPTIMAL trial reported high failure rates at 5 years (61.5% for USLS and 70.3% for SSLF), and reoperation rates of up to 30% have been described for SSLF [27, 28]. In contrast, our study demonstrated an apical recurrence rate of only 5% over 10 years, with recurrences occurring almost exclusively at the cervix rather than the vaginal vault. To our knowledge, comparable long-term data for native tissue apical suspensions are lacking. Thus, while reoperation rates may appear similar in shorter-term comparisons, the durability of apical mesh support in our series provides justification for its use, particularly given the very low incidence of vaginal vault recurrence.

### Strengths and Limitations

To our knowledge, this study represents the largest and longest follow-up retrospective investigating recurrence-associated factors following LP. Strengths include a robust follow-up duration, a large, well-defined, and homogenous patient cohort, and a single-center design that ensured consistency in surgical technique and clinical evaluation, thereby enhancing internal validity. Nonetheless, several limitations should be acknowledged. The retrospective design inherently carries risks of information and selection bias. Additionally, a notable rate of attrition over the extended follow-up period may have influenced results and limits the generalizability of the findings. These factors should be carefully considered when interpreting the study’s conclusions.

## Conclusions

This extended, long-term retrospective study confirms that, for apical prolapse as well as levels 2 and 3 defects, laparoscopic pectopexy in combination with autologous tissue reconstruction is a safe, durable, and effective option, with low recurrence and reoperation rates. The severity of the lateral defect was found to be the most important predictor of unfavorable outcomes, underscoring the importance of addressing lateral support during initial surgery. Traditional-associated factors had limited predictive value. In our study, most recurrences occurred within the first 4 years, suggesting that follow-up of at least this length is important for detecting the majority of clinically significant failures. These results support a tailored surgical approach and require further prospective studies to optimize long-term outcomes.

## Supplementary Information

Below is the link to the electronic supplementary material.

Supplementary file1 (DOCX 48 KB)

## Data Availability

Data are available on request due to privacy or ethical concerns.
