# Supplementary material for: Ten Years of Laparoscopic Pectopexy: A Case Series Analysis
Source: Int Urogynecol J. 2025 Nov 3;37(3):757–64. doi: 10.1007/s00192-025-06377-7 (PMC13032970; doi:10.1007/s00192-025-06377-7)
Supplement: Supplementary file 1 — Supplementary file1 (DOCX 48 KB) [file 192_2025_6377_MOESM1_ESM.docx]

**Supplementary Tables**

**Supplementary Table 1:** Frequency of prior gynaecological procedures among patients with a history of gynaecological surgery prior to prolapse surgery (total procedures= 502)

|  | | **Responses** | |
| --- | --- | --- | --- |
|  |  | **n** | **Percent of all procedures** |
| **Gynaecological surgeries** | Laparoscopic supracervical hysterectomy (LSH) | 70 | 13.9% |
|  | Abdominal hysterectomy | 63 | 12.5% |
|  | Vaginal hysterectomy | 121 | 24.1% |
|  | Total laparoscopic hysterectomy (TLH) | 6 | 1.2% |
|  | Abdominal supracervical hysterectomy | 2 | 0.4% |
|  | Laparoscopic Colposuspension (method Burch) | 13 | 2.6% |
|  | Abdominal Colposuspension | 13 | 2.6% |
|  | Anterior colporrhaphy | 72 | 14.3% |
|  | Posterior colporrhaphy | 59 | 11.8% |
|  | Tension-free vaginal tapes (TVT, TOT) | 14 | 2.8% |
|  | Other prolapse surgeries | 69 | 13.7% |
| **Total** | | 502 | 100.0% |

**Supplementary Table 2:** Severity Distribution of Various Types of Prolapse Among Participants

|  | **Stage 2 (N,%)** | **Stage 3 (N, %)** | **Stage 4 (N,%)** |
| --- | --- | --- | --- |
| **Uterine, cervical or vaginal vault prolapse** | 504,  60.6% | 206,  24.8% | 36,  4.3% |
| **Posterior vaginal wall prolapse** | 298,  35.8% | 54,  6.5% | 5,  0.6% |
| **Anterior vaginal wall prolapse** | 183,  22.0% | 155,  18.6% | 20,  2.4% |
| **Lateral defect*** | 283,  34.0% | 89,  10.7% | 3,  0.4% |
| *Lateral defect was evaluated clinically during pelvic examination and graded using POP-Q stage definitions for extent of descent, although this parameter is not part of the formal POP-Q system. | | | |

**Supplementary Table 3:** Frequency and Distribution of Concomitant Interventions

| **Other Interventions** | **Responses** | **Percent of patients** |
| --- | --- | --- |
| **Salpingectomy** | 374 | 44.9% |
| **Colporrhaphy anterior** | 279 | 33.5% |
| **Colporrhaphy posterior** | 374 | 44.9% |
| **Colposuspension I (Burch method)** | 63 | 7.6% |
| **Colposuspension II** | 123 | 14.8% |
| **Colposuspension III** | 140 | 16.8% |
| **Lateral repair with Burch** | 70 | 8.4% |
| **Other interventions** | 135 | 16.2% |
| Percentages refer to the proportion of patients (n=832) undergoing each concomitant intervention. Totals exceed 100% because patients could have more than one procedure. | | |

**Supplementary Table 4:** Characteristics of Participants with Recurrence or New Type of Prolapse (n=140)

| **Characteristic** | **Recurrence/De novo Group (n = 140)** |
| --- | --- |
| Age (years) | 63 (52–72) |
| Height (cm) | 165 (161–170) |
| Weight (kg) | 70 (62–80) |
| BMI (kg/m²) | 25.4 (23.1–29.3) |
| Vaginal deliveries | 2 (1–2) |
| Caesarean sections | 0 (0–0) |

**Supplementary Table 5:** Frequency and Distribution of Gynaecological Surgeries prior to prolapse surgery in patients with prolapse recurrence or de novo prolapse (n=140)

|  | | **140** | |
| --- | --- | --- | --- |
|  |  | **n** | **Percent** |
| **Gynaecological surgeries** | Laparoscopic supracervical hysterectomy (LSH) | 15 | 10.7% |
|  | Abdominal hysterectomy | 10 | 7.14% |
|  | Vaginal hysterectomy | 10 | 7.14% |
|  | Laparoscopic Colposuspension (method Burch) | 2 | 1.4% |
|  | Anterior colporrhaphy | 11 | 7.8% |
|  | Posterior colporrhaphy | 7 | 5.0% |
|  | Tension-free vaginal tapes (TVT, TOT) | 4 | 2.8% |
|  | Other prolapse surgeries | 10 | 7.14% |
| Percentages refer to the proportion of patients in the recurrence/de novo prolapse group (n=140) who had each type of prior surgery. Totals exceed 100% because some patients had more than one surgery. | | | |

**Supplementary Table 6:** Severity Distribution of Prolapse Types Among Participants with Recurrence or New Type of Prolapse (n=140)

|  | **Stage 2 (N,%)** | **Stage 3 (N, %)** | **Stage 4 (N,%)** |
| --- | --- | --- | --- |
| **Uterine, cervical or vaginal vault prolapse** | 88,  62.9% | 41,  29.3% | 6,  4.3% |
| **Posterior vaginal wall prolapse** | 45,  32.1% | 11,  7.9% | 1,  0.7% |
| **Anterior vaginal wall prolapse** | 31,  22.1% | 25,  17.9% | 7,  5.0% |
| **Lateral defect*** | 54,  38.6% | 31,  22.1% | 2,  1.4% |
| *Lateral defect was evaluated clinically during pelvic examination and graded using POP-Q stage thresholds for extent of descent, although this parameter is not part of the formal POP-Q system. | | | |

**Supplementary Table 7:** Compartment-Specific Recurrence and De Novo POP (n = 832)

| **Compartment / Outcome** | **Count (n)** | **% of Total Cohort** |
| --- | --- | --- |
| **Recurrence** |  |  |
| Apical recurrence | 67 | 8.0% |
| Anterior vaginal wall recurrence | 17 | 2.0% |
| Lateral defect recurrence | 11 | 1.3% |
| Posterior vaginal wall recurrence | 18 | 2.2% |
| Re-surgery at first presentation | 53 | 6.4% |
| **De Novo POP** |  |  |
| De novo anterior vaginal wall prolapse | 50 | 6.0% |
| De novo lateral defect | 25 | 3.0% |
| De novo posterior vaginal wall prolapse | 36 | 4.3% |
| Surgery for de novo defects | 60 | 7.2% |

**Supplementary Table 8:** Comparison of Characteristics Between Groups with (n = 67) and without (n = 765) Apical Recurrence during Follow-Up

|  | **Median (No Recurrence)** | **IQR (No Recurrence)** | **Median (Recurrence)** | **IQR (Recurrence)** | **Mann–Whitney U** | **p-value** |
| --- | --- | --- | --- | --- | --- | --- |
| **Age (years)** | 63.0 | 20.00 | 63.0 | 20.50 | 24920.5 | 0.734 |
| **BMI (kg/m²)** | 25.76 | 5.77 | 25.56 | 5.65 | 22204.5 | 0.401 |
| **Number of vaginal deliveries** | 2 | 1.00 | 2 | 1.00 | 21927 | 0.168 |
| **Number of caesarean sections** | 0 | 0.00 | 0 | 0.00 | 21964 | 0.128 |
| **Uterine, cervical or vaginal vault prolapse** | 2 | 1.00 | 2 | 1.00 | 27981.5 | 0.153 |
| **Posterior vaginal wall prolapse** | 1 | 2.00 | 1 | 2.00 | 27027.5 | 0.435 |
| **Anterior vaginal wall prolapse** | 1 | 2.00 | 0 | 2.00 | 24456.5 | 0.509 |
| **Lateral defect** | 1 | 2.00 | 2 | 1.50 | 31416.5 | <0.001 |
| IQR: Interquartile Range, p: p-value | | | | | | |

**Supplementary Table 9:** Comparison of Characteristics Between Groups with (n = 53) and without (n = 779) Re-surgery during follow-up

|  | **Median (No Re-surgery)** | **IQR (No Re-surgery)** | **Median (Re-surgery)** | **IQR (Re-surgery)** | **Mann–Whitney U** | **p-value** |
| --- | --- | --- | --- | --- | --- | --- |
| **Age (years)** | 63.0 | 20.00 | 65.0 | 22.00 | 21792 | 0.477 |
| **BMI (kg/m²)** | 25.71 | 5.71 | 26.27 | 6.29 | 20078 | 0.346 |
| **Number of vaginal deliveries** | 2 | 1.00 | 2 | 1.00 | 18099.5 | 0.657 |
| **Number of caesarean sections** | 0 | 0.00 | 0 | 0.00 | 17374 | 0.289 |
| **Uterine, cervical or vaginal vault prolapse** | 2 | 1.00 | 2 | 1.00 | 23177.5 | 0.086 |
| **Posterior vaginal wall prolapse** | 1 | 2.00 | 2 | 2.00 | 23413.5 | 0.085 |
| **Anterior vaginal wall prolapse** | 1 | 2.00 | 0 | 2.00 | 20137 | 0.750 |
| **Lateral defect** | 1 | 2.00 | 2 | 1.00 | 27133 | <0.001 |
| IQR: Interquartile Range, p: p-value | | | | | | |

**Supplementary Table 10:** Comparison of Characteristics Between Groups with (n = 60) and without (n = 772) surgery due to de novo prolapse during follow-up

|  | **Median (No Re-surgery)** | **IQR (No Re-surgery)** | **Median (Re-surgery)** | **IQR (Re-surgery)** | **Mann–Whitney U** | **p-value** |
| --- | --- | --- | --- | --- | --- | --- |
| **Age (years)** | 63.0 | 20.00 | 63.0 | 21.25 | 23068.5 | 0.986 |
| **BMI (kg/m²)** | 25.68 | 5.68 | 26.44 | 6.22 | 24424 | 0.067 |
| **Number of vaginal deliveries** | 2 | 1.00 | 2 | 1.00 | 22807 | 0.295 |
| **Number of caesarean sections** | 0 | 0.00 | 0 | 0.00 | 20240 | 0.410 |
| **Uterine, cervical or vaginal vault prolapse** | 2 | 1.00 | 2 | 1.00 | 25584 | 0.122 |
| **Posterior vaginal wall prolapse** | 1 | 2.00 | 1 | 2.00 | 21587 | 0.356 |
| **Anterior vaginal wall prolapse** | 1 | 2.00 | 0 | 2.00 | 21417 | 0.301 |
| **Lateral defect** | 1 | 2.00 | 2 | 2.00 | 31080.5 | <0.001 |
| IQR: Interquartile Range, p: p-value | | | | | | |

**Supplementary Table 11:** Association Between Hysterectomy Types and Apical Recurrence at Follow-Up

|  | | **Apical recurrence 1^st^ follow-up** | | | | | |
| --- | --- | --- | --- | --- | --- | --- | --- |
|  |  | ***No*** | | ***Yes*** | | ***X^2^*** | ***p*** |
|  |  | *n* | *%* | *n* | *%* |  |  |
| **Laparoscopic supracervical hysterectomy (LSH)** | *No* | 240 | 79.2% | 12 | 63.2% | 2.707 | 0.146 ^f^ |
|  | *Yes* | 63 | 20.8% | 7 | 36.8% |  |  |
| **Abdominal hysterectomy** | *No* | 243 | 80.2% | 16 | 84.2% | 0.183 | 1.000 ^f^ |
|  | *Yes* | 60 | 19.8% | 3 | 15.8% |  |  |
| **Vaginal hysterectomy** | *No* | 183 | 60.4% | 18 | 94.7% | 8.989 | **0.003** |
|  | *Yes* | 120 | 39.6% | 1 | 5.3% |  |  |
| **Total laparoscopic hysterectomy (TLH)** | *No* | 297 | 98.0% | 19 | 100.0% | 0.383 | 1.000 ^f^ |
|  | *Yes* | 6 | 2.0% | 0 | 0.0% |  |  |
| **Abdominal supracervical hysterectomy** | *No* | 301 | 99.3% | 19 | 100.0% | 0.126 | 1.000^f^ |
|  | *Yes* | 2 | 0.7% | 0 | 0.0% |  |  |
| ^f^  Fisher’s Exact Test, c, x^2^: Chi-square statistic, p: p-value, n: sample size | | | | | | | |

**Supplementary Table 12:** Logistic regression analysis investigating factors associated with recurrence or the development of a new type of prolapse

| *Variables in the Equation* | | | | | | | | | |
| --- | --- | --- | --- | --- | --- | --- | --- | --- | --- |
|  | | B | S.E. | Wald | df | p | OR | 95% C.I.for OR | |
|  |  |  |  |  |  |  |  | Lower | Upper |
| Step 1^a^ | Age | .006 | .009 | .383 | 1 | .536 | 1.006 | .988 | 1.024 |
|  | BMI | .001 | .022 | .002 | 1 | .961 | 1.001 | .960 | 1.044 |
|  | Number of vaginal deliveries | -.050 | .102 | .242 | 1 | .623 | .951 | .780 | 1.161 |
|  | Number of caesarean sections | -.311 | .343 | .824 | 1 | .364 | .733 | .374 | 1.434 |
|  | Uterine, cervical or vaginal vault prolapse | .125 | .134 | .865 | 1 | .352 | 1.133 | .871 | 1.474 |
|  | Posterior vaginal wall prolapse | -.002 | .104 | .000 | 1 | .988 | .998 | .814 | 1.225 |
|  | Anterior vaginal wall prolapse | .041 | .085 | .237 | 1 | .626 | 1.042 | .882 | 1.231 |
|  | Lateral defect | .439 | .092 | 22.656 | 1 | <.001 | 1.551 | 1.294 | 1.858 |
|  | Gynecological surgeries(1) | -.235 | .215 | 1.192 | 1 | .275 | .791 | .518 | 1.205 |
|  | Constant | -2.655 | .868 | 9.363 | 1 | .002 | .070 |  |  |
| B: Regression Coefficient (Beta), S.E.: Standard Error, Wald: Wald Chi-Square Statistic, df: Degrees of Freedom, p: p-value, OR: Odds Ratio, 95% C.I.for OR: 95% Confidence Interval for Odds Ratio | | | | | | | | | |

**Supplementary Table 13:** Logistic regression analysis investigating factors associated with the need for re-surgery at the first follow-up

| *Variables in the Equation* | | | | | | | | | |
| --- | --- | --- | --- | --- | --- | --- | --- | --- | --- |
|  | | B | S.E. | Wald | df | p | OR | 95% C.I.for OR | |
|  |  |  |  |  |  |  |  | Lower | Upper |
| Step 1^a^ | Age | .026 | .014 | 3.252 | 1 | .071 | 1.026 | .998 | 1.055 |
|  | BMI | .044 | .032 | 1.883 | 1 | .170 | 1.045 | .981 | 1.113 |
|  | Number of vaginal deliveries | -.054 | .154 | .123 | 1 | .726 | .948 | .701 | 1.280 |
|  | Number of caesarean sections | -.469 | .613 | .585 | 1 | .444 | .626 | .188 | 2.081 |
|  | Uterine, cervical or vaginal vault prolapse | .091 | .197 | .211 | 1 | .646 | 1.095 | .744 | 1.611 |
|  | Posterior vaginal wall prolapse | .330 | .166 | 3.942 | 1 | .047 | 1.391 | 1.004 | 1.925 |
|  | Anterior vaginal wall prolapse | -.083 | .132 | .401 | 1 | .527 | .920 | .711 | 1.191 |
|  | Lateral defect | .581 | .144 | 16.186 | 1 | <.001 | 1.787 | 1.347 | 2.371 |
|  | Gynecological surgeries(1) | -.572 | .351 | 2.654 | 1 | .103 | .564 | .284 | 1.123 |
|  | Constant | -6.526 | 1.394 | 21.908 | 1 | <.001 | .001 |  |  |
| B: Regression Coefficient (Beta), S.E.: Standard Error, Wald: Wald Chi-Square Statistic, df: Degrees of Freedom, p: p-value, OR: Odds Ratio, 95% C.I.for OR: 95% Confidence Interval for Odds Ratio | | | | | | | | | |

**Supplementary Table 14:** Logistic regression analysis investigated factors associated with apical recurrence at the first follow-up

| *Variables in the Equation* | | | | | | | | | |
| --- | --- | --- | --- | --- | --- | --- | --- | --- | --- |
|  | | B | S.E. | Wald | df | p | OR | 95% C.I.for OR | |
|  |  |  |  |  |  |  |  | Lower | Upper |
| Step 1^a^ | Age | .007 | .026 | .077 | 1 | .781 | 1.007 | .957 | 1.060 |
|  | BMI | -.042 | .065 | .411 | 1 | .521 | .959 | .845 | 1.089 |
|  | Number of vaginal deliveries | -.317 | .286 | 1.234 | 1 | .267 | .728 | .416 | 1.274 |
|  | Number of caesarean sections | -18.554 | 6184.357 | .000 | 1 | .998 | .000 | .000 | . |
|  | Uterine, cervical or vaginal vault prolapse | .023 | .319 | .005 | 1 | .942 | 1.023 | .547 | 1.913 |
|  | Posterior vaginal wall prolapse | .177 | .254 | .483 | 1 | .487 | 1.193 | .725 | 1.963 |
|  | Anterior vaginal wall prolapse | -.070 | .237 | .088 | 1 | .767 | .932 | .586 | 1.483 |
|  | Lateral defect | .287 | .228 | 1.578 | 1 | .209 | 1.332 | .852 | 2.083 |
|  | Vaginal hysterectomy(1) | -2.447 | 1.054 | 5.389 | 1 | .020 | .087 | .011 | .683 |
|  | Constant | -1.461 | 2.530 | .333 | 1 | .564 | .232 |  |  |
| B: Regression Coefficient (Beta), S.E.: Standard Error, Wald: Wald Chi-Square Statistic, df: Degrees of Freedom, p: p-value, OR: Odds Ratio, 95% C.I.for OR: 95% Confidence Interval for Odds Ratio | | | | | | | | | |

**Supplementary Table 15:** Logistic regression analysis investigated factors associated with the likelihood of requiring surgery at the first follow-up

| *Variables in the Equation* | | | | | | | | | |
| --- | --- | --- | --- | --- | --- | --- | --- | --- | --- |
|  | | B | S.E. | Wald | df | p | OR | 95% C.I.for OR | |
|  |  |  |  |  |  |  |  | Lower | Upper |
| Step 1^a^ | Age | .020 | .013 | 2.233 | 1 | .135 | 1.020 | .994 | 1.047 |
|  | BMI | .063 | .029 | 4.831 | 1 | .028 | 1.065 | 1.007 | 1.127 |
|  | Number of vaginal deliveries | .166 | .135 | 1.518 | 1 | .218 | 1.180 | .907 | 1.537 |
|  | Number of caesarean sections | -.312 | .522 | .357 | 1 | .550 | .732 | .263 | 2.037 |
|  | Uterine, cervical or vaginal vault prolapse | .077 | .198 | .152 | 1 | .697 | 1.080 | .732 | 1.594 |
|  | Posterior vaginal wall prolapse | -.124 | .160 | .604 | 1 | .437 | .883 | .646 | 1.208 |
|  | Anterior vaginal wall prolapse | -.134 | .128 | 1.089 | 1 | .297 | .875 | .681 | 1.124 |
|  | Lateral defect | .663 | .143 | 21.590 | 1 | <.001 | 1.940 | 1.467 | 2.565 |
|  | Gynecological surgeries(1) | -.708 | .342 | 4.287 | 1 | .038 | .493 | .252 | .963 |
|  | Constant | -6.451 | 1.271 | 25.758 | 1 | <.001 | .002 |  |  |
| B: Regression Coefficient (Beta), S.E.: Standard Error, Wald: Wald Chi-Square Statistic, df: Degrees of Freedom, p: p-value, OR: Odds Ratio, 95% C.I.for OR: 95% Confidence Interval for Odds Ratio | | | | | | | | | |
